# Epigallocatechin gallate restores the reduction of protein phosphatase 2 A subunit B caused by middle cerebral artery occlusion

**DOI:** 10.1186/s42826-023-00155-6

**Published:** 2023-02-14

**Authors:** Murad-Ali Shah, Ju-Bin Kang, Dong-Ju Park, Phil Ok Koh

**Affiliations:** Department of Anatomy, College of Veterinary Medicine, Research Institute of Life Science, Gyeongsang National University, 501 Jinjudaero, 52828 Jinju, South Korea

**Keywords:** Cerebral ischemia, Epigallocatechin gallate, Neuroprotection, Protein phosphatase 2A

## Abstract

**Background:**

Epigallocatechin gallate (EGCG) is a flavonoid compound commonly found in green tea. It exhibits antioxidant, anti-inflammatory, and neuroprotective effects in cerebral ischemia. Protein phosphatase 2 A (PP2A) is an important serine/threonine phosphatase enzyme involved in various cellular activities. PP2A subunit B is present abundantly in the brain and plays an important role in the nervous system. We investigated the effect of EGCG on the expression level of PP2A subunit B in cerebral ischemia caused by middle cerebral artery occlusion (MCAO). EGCG (50 mg/kg) or vehicle was injected into the peritoneal cavity prior to MCAO surgery. Neurological behavior tests were performed 24 h after MCAO, and right cerebral cortex tissue was collected. Cerebral ischemia caused serious neurological abnormalities, which were alleviated by EGCG administration. We screened the expression of PP2A subunits containing A, B, and C using reverse-transcription PCR. We confirmed that PP2A subunit B exhibited significant changes in MCAO animals compared to subunits A and C. We continuously examined the expression of PP2A subunit B protein in MCAO animals using Western blot analysis.

**Results:**

EGCG alleviated the reduction of PP2A subunit B protein by MCAO damage. In addition, immunohistochemistry demonstrated a decrease in the number of PP2A subunit B-positive cells in the cerebral cortex, and EGCG attenuated this decrease. Maintenance of PP2A subunit B is important for normal brain function.

**Conclusion:**

Therefore, our findings suggest that EGCG exerts neuroprotective effects against cerebral ischemia through modulation of PP2A subunit B expression.

## Background

Epigallocatechin gallate (EGCG) is a flavonoid found abundantly in green tea [1]. It exhibits the most potent antioxidant activity among the many types of catechins present in green tea. EGCG is more effective in radical scavenging compared to vitamins C and E [2, 3]. In addition, EGCG alleviates the inflammatory response and improves neurological disorders against ischemic damage [4]. It has the advantage of easily passing through the blood–brain barrier even at very low concentrations [5]. EGCG has a powerful neuroprotective ability against cerebral ischemia, prevents the production of reactive oxygen species (ROS), and reduces infarction volume and neurological disorders [6, 7]. Moreover, EGCG alleviates cognitive impairment and neurobehavioral disorders in Alzheimer’s and Parkinson’s models, respectively [8, 9].

Cerebral ischemia is caused by clogging of cerebral blood flow, which interferes with the supply of oxygen and nutrients to the brain. Since the brain lacks oxygen storage, even if it is a few seconds of ischemia, ischemia can cause complete unconsciousness and result in serious brain damage [10]. The lack of oxygen and glucose in the brain causes serious pathophysiological and neurological disorders. It also activates inflammatory mediators and leads to inflammation in the ischemic region of the brain [11]. Ischemia is one of the major causes of production of ROS that can induce cell death [12]. It also induces apoptosis through activation of exogenous and endogenous apoptosis pathways [13].

Protein phosphatase 2 A (PP2A) is a serine/threonine phosphatase enzyme associated with various cellular functions. PP2A plays an important role in the regulation of the cell cycle, cell growth and development, signal transduction pathways, and cell mobility [14–16]. PP2A is composed of structural A, regulatory B, and catalytic C subunits. Subunits A and C form core enzymes through their binding, and the regulatory B subunit is attached to the core enzymes to form holoenzymes [17]. PP2A is found ubiquitously in all cells and accounts for 0.3–1% of all cell proteins [18]. Catalytic C subunits are most abundantly expressed among PP2A subunits. Subunits A and C exist in various tissues, whereas subunit B is specifically observed in brain tissues. In addition, subunit B contributes to neuronal development and axonal outgrowth in the brain [19]. Therefore, PP2A subunit B is considered an important factor in neuroprotective mechanisms against brain damage. We also expect that EGCG regulates PP2A expression in cerebral ischemia. Although the neuroprotective effect of EGCG has been revealed, the mechanism is not fully understood. Furthermore, studies on regulation of PP2A subunit B by EGCG have not been reported. The purpose of this study was to investigate the regulation of PP2A subunit B expression by EGCG administration in ischemic brain injury.

## Results

### Improvement of neurobehavioral disturbance by EGCG treatment in MCAO injury

**Fig. 1:**
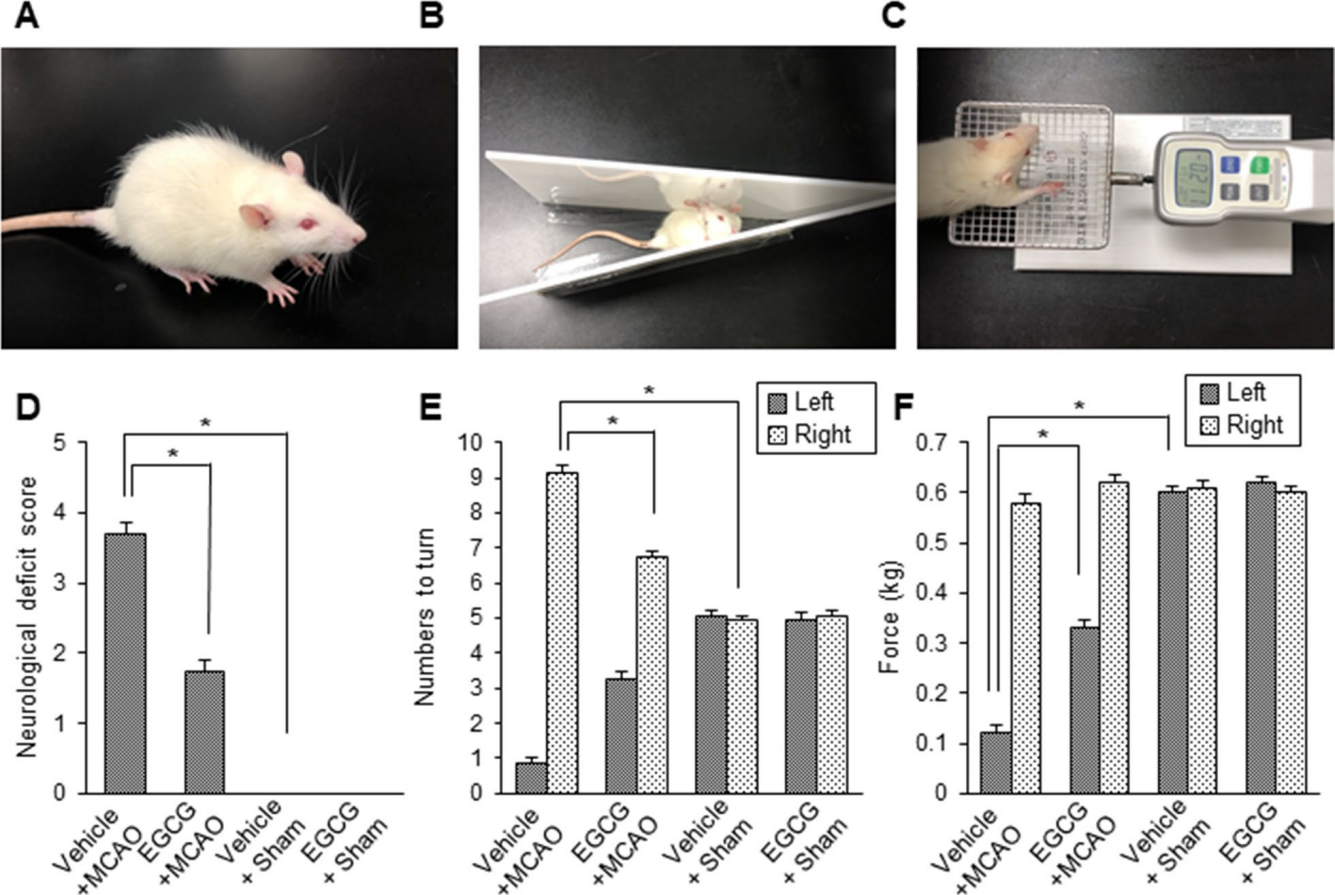
EGCG improves neurobehavioral disorders caused by cerebral ischemia. Representative images and graphs of neurobehavioral scoring test (**A**, **D**) corner test (**B**, **E**), and grip strength test (**C**, **F**) in the right cerebral cortex of vehicle + middle cerebral artery occlusion (MCAO), EGCG + MCAO, vehicle + sham, and EGCG + sham animals. EGCG improved the neurobehavioral deficits induced by MCAO injury. Data (*n* = 16) are presented as mean ± S.E.M. *p < 0.05 vs. vehicle + MCAO animals

Cerebral ischemia caused by middle cerebral artery occlusion (MCAO) surgery results in serious neurological deficits and neuropathological lesions. We confirmed these changes through a variety of neurological behavioral tests, including neurological deficits scoring tests (Fig. 1A), corner tests (Fig. 1B), and grip strength tests (Fig. 1C). MCAO damage leads to severe neurological disorders, which can be alleviated by EGCG treatment. The result of neurological deficits scoring tests showed severe neurological deficits in the MCAO animals treated with vehicle. Some animals circled repeatedly to the one side, others showed seizures or unconsciousness. However, EGCG treatment alleviated these deficits. The neurological deficits score was 3.71 ± 0.15 in the vehicle + MCAO animals and 1.75 ± 0.16 in the EGCG + MCAO animals (Fig. 1D). We found no neurological deficits in the vehicle + sham and EGCG + sham animals. The results of the corner test showed an increase in the number of right turns for MCAO animals treated with vehicle. EGCG treatment attenuated this increase caused by MCAO damage. The right MCAO animal is less responsive to right stimuli due to the right cerebral cortex damage. Thus, the sensitivity of the right vibrissae decreases, causing the animal to turn to the right side. The number of right turns was 9.16 ± 0.18 in the vehicle + MCAO and 6.72 ± 0.19 in the EGCG + MCAO animals. The number of left turns was 0.84 ± 0.18 and 3.28 ± 0.19 in the vehicle + MCAO and EGCG + MCAO animals, respectively (Fig. 1E). Sham animals showed a similar number of right and left turns regardless of vehicle and EGCG administration. The grip strength of the left forelimb was significantly decreased in MCAO animals treated with vehicle. This decrease is due to damage to the right cerebral cortex. However, EGCG treatment significantly improved the grip strength in the left forelimb. The grip strength for the left forelimbs was 0.12 ± 0.02 in the vehicle + MCAO and 0.33 ± 0.02 in EGCG + MCAO animals. The grip strength for the right forelimbs was 0.58 ± 0.02 and 0.62 ± 0.02 in the vehicle + MCAO and EGCG + MCAO animals, respectively (Fig. 1F). Sham animals had similar levels of grip strength in both left and right forelimbs.

### Improvement of neuropathological changes by EGCG treatment in MCAO damage

**Fig. 2:**
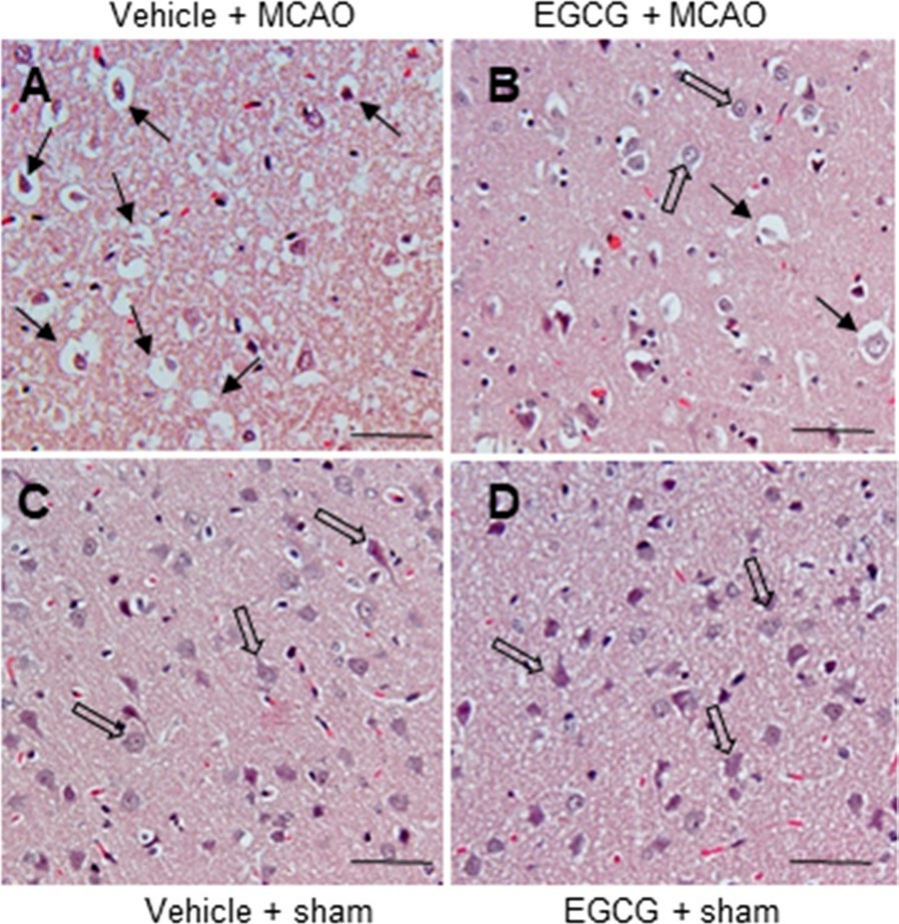
EGCG alleviates neuropathological changes caused by cerebral ischemia. Images of hematoxylin and eosin staining (**A**–**D**) from the right cerebral cortex of vehicle + middle cerebral artery occlusion (MCAO), EGCG + MCAO, vehicle + sham, and EGCG + sham animals. EGCG treatment alleviated the histopathological changes caused by MCAO damage. Arrows represent abnormal cells that show serious histopathological changes. Open arrows represent typical normal neurons. Scale bar = 100 μm

**Fig. 3:**
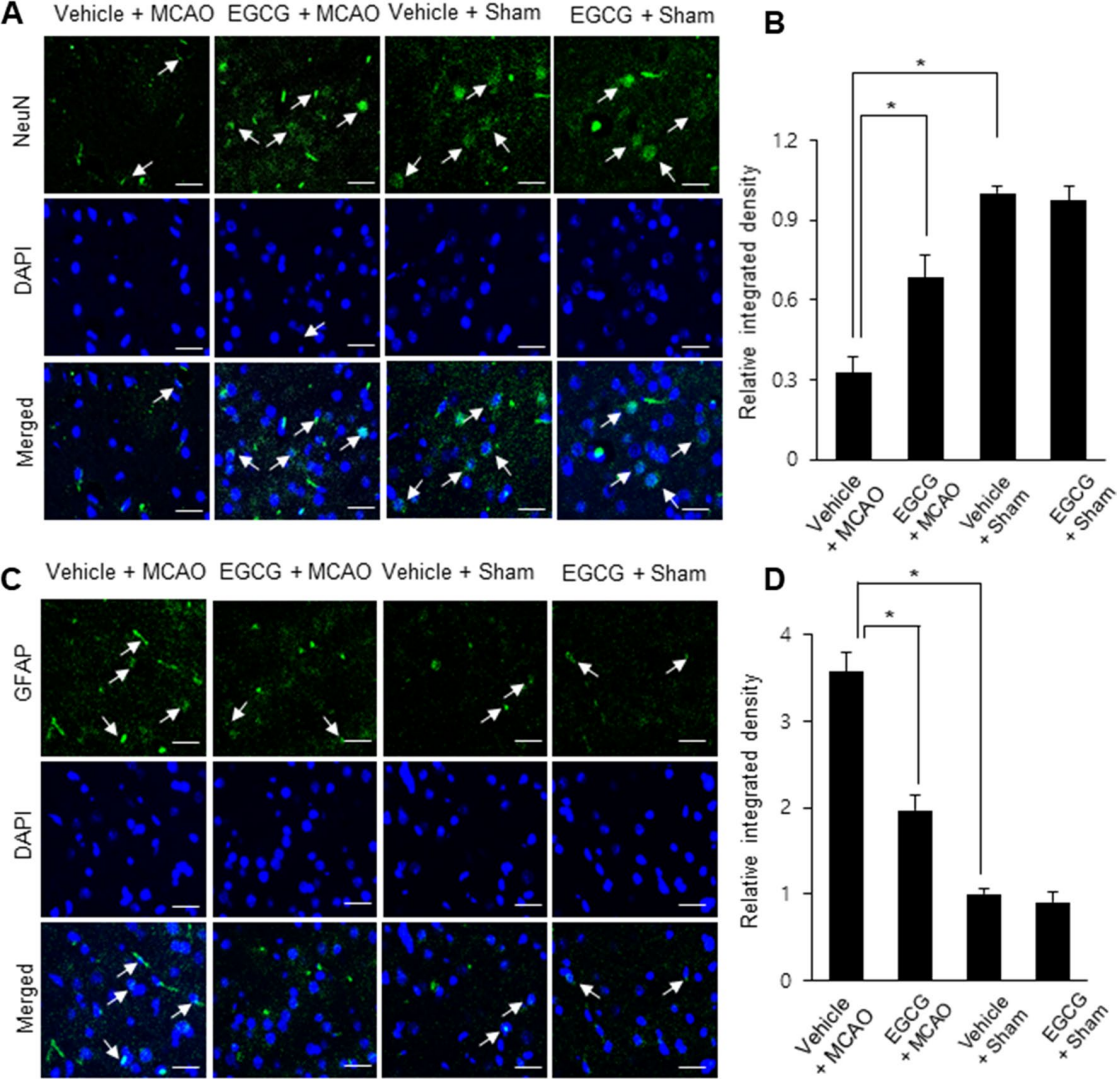
EGCG attenuates changes in NeuN and GFAP immunoreactivity caused by cerebral ischemia. Representative images and graphs for the immunofluorescence staining of NeuN (**A**, **B**) and GFAP (**C**, **D**) in the right cerebral cortex of vehicle + middle cerebral artery occlusion (MCAO), EGCG + MCAO, vehicle + sham, and EGCG + sham animals. Arrows indicate NeuN- and GFAP-positive reactions. The intensity values of NeuN and GFAP immunoreactivity are expressed as the ratio of the intensity of each group to the intensity of vehicle + sham animals. The level of vehicle + sham animals was set to 1. Data (*n* = 4) are represented as the mean ± S.E.M. *p < 0.05 vs. vehicle + MCAO animals. Scale bar = 50 μm

Results of hematoxylin and eosin staining showed severe histopathological changes in the cerebral cortex of MCAO animals with vehicle (Fig. 2A). These animals had neurons with abnormal shapes, including shrunken nuclei, intracellular vacuole, expansion, and dendritic loss. However, these changes were mitigated by EGCG treatment (Fig. 2B). Sham animals regardless of vehicle or EGCG treatment had neurons with a typical pyramidal shape, including large nuclei and well-developed dendrites (Fig. 2C, D). The results of immunofluorescence staining revealed changes in the immunoreactivity of neuronal nuclear protein (NeuN) and glial fibrillary acidic protein (GFAP) in the cerebral cortex of vehicle-treated MCAO animals. In MCAO animals, NeuN immunoreactivity was decreased and GFAP immunoreactivity was increased. However, EGCG treatment ameliorated these changes caused by MCAO damage 9 (Fig. 3A, C). The level of NeuN immunoreactivity was 0.32 ± 0.05 in the vehicle + MCAO and 0.68 ± 0.08 in the EGCG + MCAO animals (Fig. 3B). The level of GFAP immunoreactivity was 3.57 ± 0.23 and 1.96 ± 0.17 in the vehicle + MCAO and EGCG + MCAO animals, respectively (Fig. 3D).

### Mitigation of PP2A subunits mRNA levels reduction by EGCG administration in MCAO damage

**Fig. 4:**
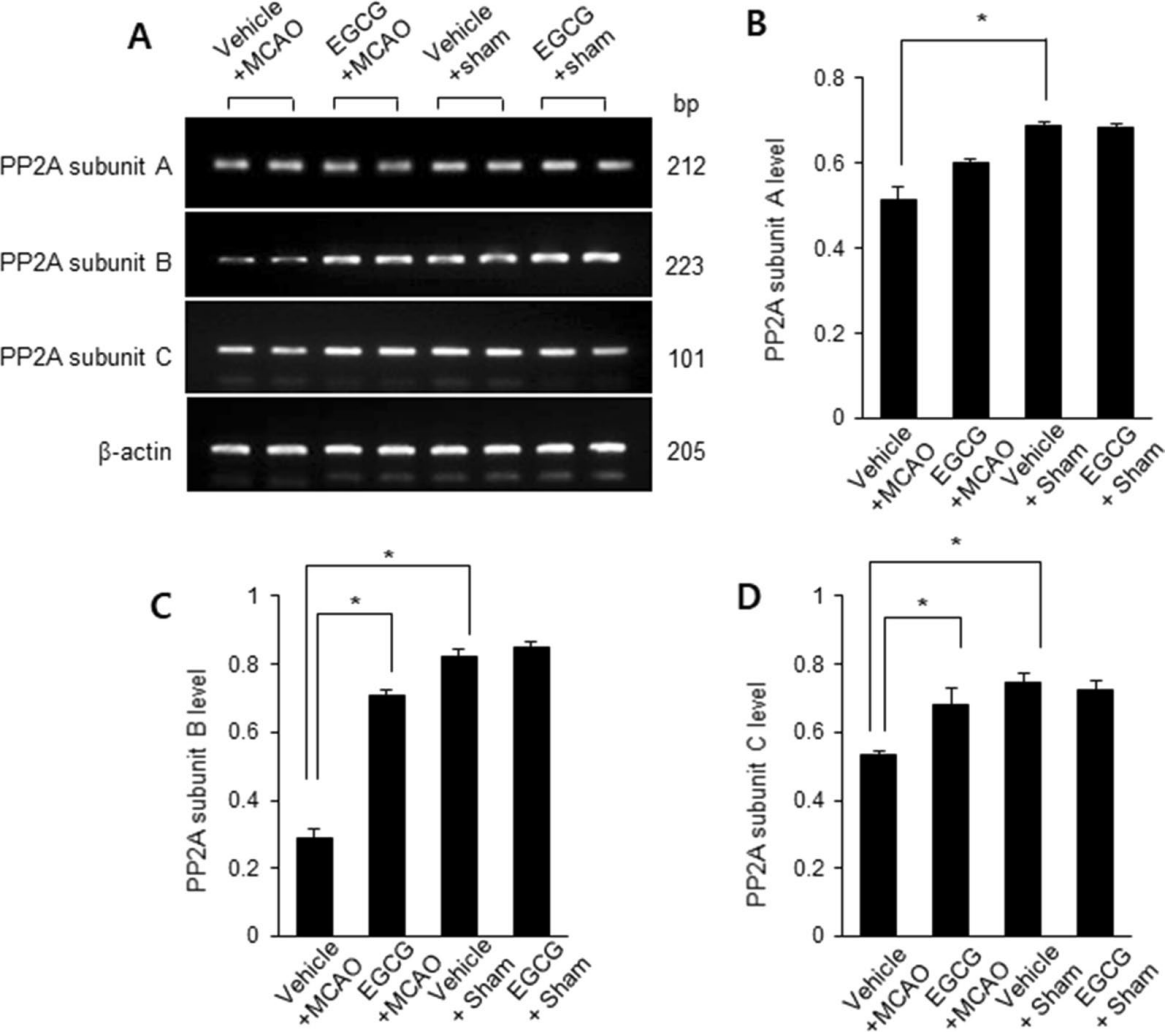
EGCG alleviates the decrease in protein phosphatase 2 A (PP2A) subunits mRNA expressions induced by cerebral ischemia. Reverse transcription PCR for PP2A in the right cerebral cortex of vehicle + middle cerebral artery occlusion (MCAO), EGCG + MCAO, vehicle + sham, and EGCG + sham animals. Each band represents the results of transcription for PP2A in individual animals (**A**). The level of PP2A mRNA was expressed as the ratio of the intensity of the PCR product to the intensity of β-actin (**B**–**D**). Data (*n* = 4) are represented as the mean ± S.E.M. *p < 0.05 vs. vehicle + MCAO animals

We observed the transcription levels of PP2A subunits A, B, and C in the ischemic right cerebral cortex. Reverse transcription PCR was carried out to elucidate these changes. The transcription levels of all PP2A subunits were reduced in MCAO animals with a vehicle, and EGCG treatment attenuated these reductions (Fig. 4A). Among the PP2A subunits, the expression of B subunit was significantly altered compared to subunits A and C. The transcription level of PP2A subunit A was 0.51 ± 0.03 in the vehicle + MCAO and 0.60 ± 0.01 in the EGCG + MCAO animals (Fig. 4B). The level of PP2A subunit B protein was 0.31 ± 0.02 and 0.76 ± 0.07 in the vehicle + MCAO and EGCG + MCAO animals, respectively (Fig. 4C). The level of subunit C was 0.53 ± 0.01 in the vehicle + MCAO and 0.68 ± 0.05 in the EGCG + MCAO animals (Fig. 4D). The expression of all subunits in sham-operated animals did not change regardless of vehicle or EGCG treatment. The expression of PP2A subunit B mRNA was the most changed among subunits, so this study focused on the expression of PP2A subunit B.

### Alleviation of PP2A subunit B protein level reduction by EGCG administration in MCAO damage

**Fig. 5:**
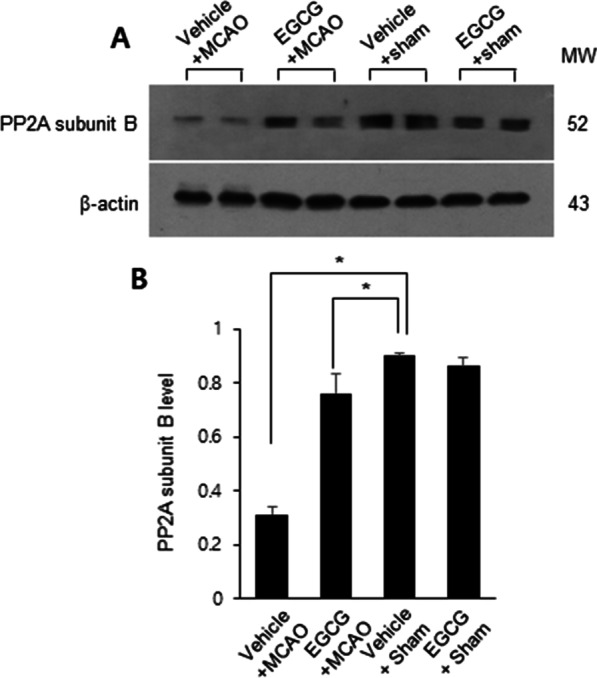
EGCG alleviates the decrease in protein phosphatase 2 A (PP2A) subunit B protein expressions induced by cerebral ischemia. Western blot analysis for the PP2A subunit B protein in the right cerebral cortex of vehicle + middle cerebral artery occlusion (MCAO), EGCG + MCAO, vehicle + sham, and EGCG + sham animals. Each band represents the results of Western blot analysis in individual animals (**A**). The level of PP2A protein was expressed as the ratio of the intensity of the PP2A band to the intensity of the β-actin band (**B**). Data (*n* = 4) are represented as the mean ± S.E.M. *p < 0.05 vs. vehicle + MCAO animals

**Fig. 6:**
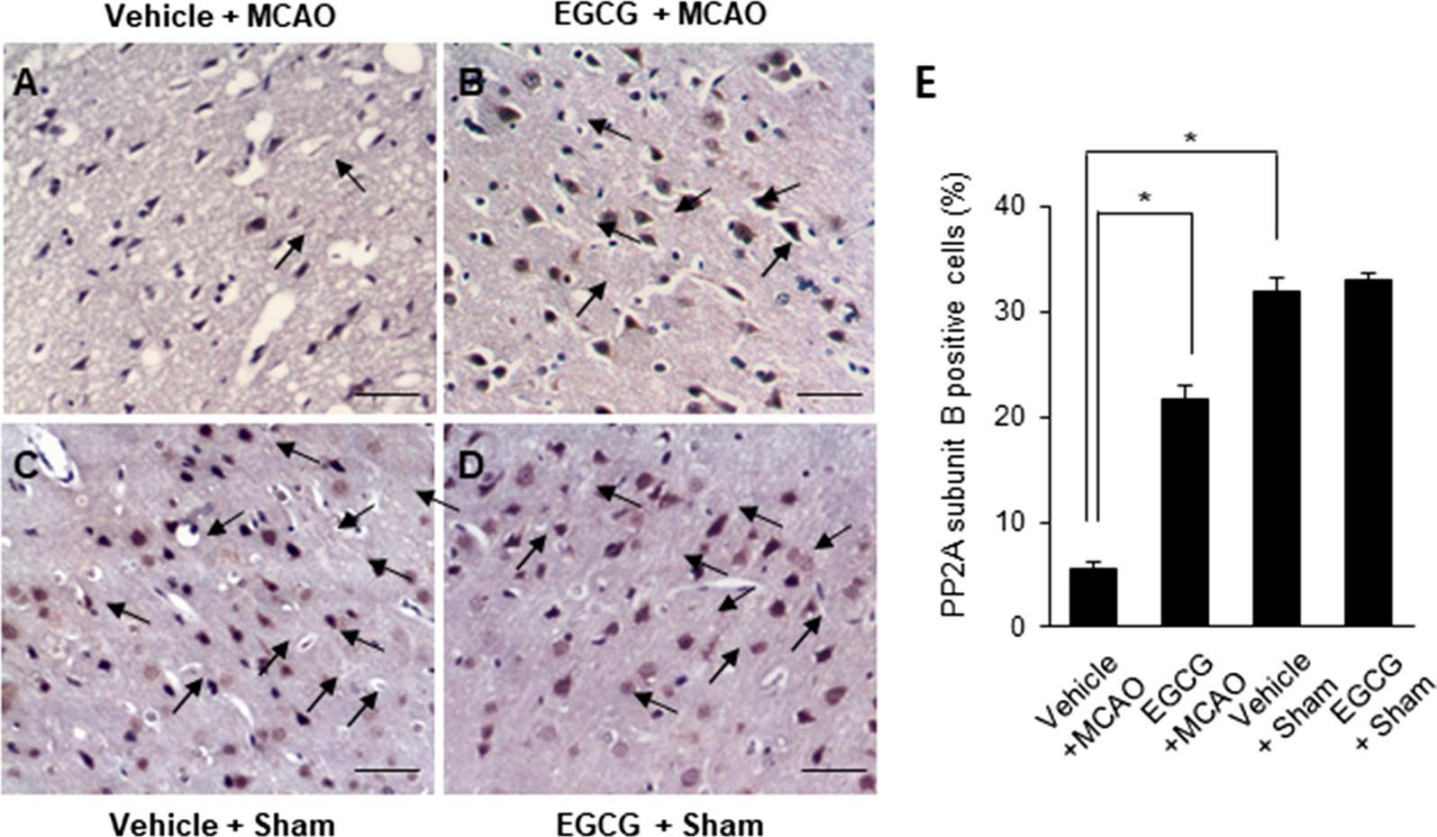
EGCG attenuates the decrease in the number of protein phosphatase 2 A (PP2A) subunit B-positive cells caused by cerebral ischemia. Images of the immunohistochemical staining of PP2A in the right cerebral cortex of vehicle + middle cerebral artery occlusion (MCAO) (**A**), EGCG + MCAO (**B**), vehicle + sham (**C**), and EGCG + sham (**D**) animals. Arrows indicate PP2A subunit B-positive cells. The expression level of PP2A subunit B is presented as the percentage of the number of PP2A subunit B-positive cells to the total number of neurons (**E**). Data (*n* = 4) are represented as the mean ± S.E.M. *p < 0.05 vs. vehicle + MCAO animals. Scale bar = 100 μm

We observed the expression of PP2A subunit B protein in succession. The results of Western blot analysis showed the decrease in PP2A subunit B proteins in MCAO animals and alleviation of these decreases by EGCG treatment (Fig. 5A). The level of PP2A subunit B protein was 0.31 ± 0.02 and 0.76 ± 0.07 in the vehicle + MCAO and EGCG + MCAO animals, respectively (Fig. 5B). Immunohistochemical staining showed that PP2A subunit B positive reaction decreased in MCAO animals (Fig. 6A). In addition, we observed the mitigation of these reductions in the presence of EGCG (Fig. 6B). The number of PP2A subunit B positive cells in sham animals were similar regardless of vehicle or EGCG treatment (Fig. 6C, D). We counted the number of PP2A subunit B-positive cells in the cerebral cortex region. The number of PP2A subunit B-positive cells was 5.61 ± 0.57 in the vehicle + MCAO and 21.65 ± 1.31 in the EGCG + MCAO animals (Fig. 6E).

## Discussion

EGCG is a flavonoid with strong antioxidant and radical scavenging properties [2, 3]. It plays a role in neuroprotection against oxidative stress through ROS removal [20]. EGCG also has potent anti-inflammatory and neuroprotective properties, improving neurological disorders, reducing infarction volumes, and protecting neurons from cerebral ischemia [4, 6, 7, 21–23]. We have previously shown that EGCG exerts neuroprotective effects in focal cerebral ischemia [24, 25]. We also have demonstrated that EGCG plays a neuroprotective role by removing ROS and lipid peroxidation and by regulating antioxidant proteins such as thioredoxin [25]. We found severe neurologic deficits in MCAO animals using a variety of neurobehavioral tests such as neurologic deficit score tests, corner tests, and grip strength tests. However, EGCG treatment improved these behavioral disturbances caused by MCAO damage and exerted neuroprotective effects against brain damage. These findings were consistent with previous studies on the neuroprotective effects of EGCG [24, 25]. These results provide evidence that EGCG can prevent neurological disorders caused by cerebral ischemia. We also found severe histopathological changes caused by MCAO damage. EGCG treatment alleviates these pathological lesions. We tried immunohistochemical staining for the detection of neurons and astrocytes. NeuN and GFAP were used as markers of neurons and astrocytes, respectively. MCAO damage decreased the immunoreactivity of NeuN and increased the immunoreactivity of GFAP, and EGCG treatment prevented these changes. The decline of NeuN indicates a decrease in the number of neurons, and the increase of GFAP indicates the activation of astrocytes. Astrocytes are known as the major glial cells of the central nervous system. Because nerve damage activates astrocytes and rapidly elevates GFAP production, GFAP levels are utilized as markers of neurological damage in trauma and stroke [26]. These results demonstrate that ischemic brain damage affects both neurons and astrocytes, causing histopathological change and cell damage. Moreover, EGCG attenuates cell damage to ischemia and has strong neuroprotective properties.

We further explored EGCG regulation of the expression of PP2A subunit B in MCAO-induced cerebral ischemia. We identified the decrease of PP2A including subunits A, B, and C in MCAO animals. These findings were screened using reverse transcription PCR techniques. Although subunits A and C were significantly changed caused by MCAO damage and the change of these subunits by EGCG administration was significant, the change of subunit B by EGCG administration was more significant than those of subunits A and C. We investigated whether EGCG regulates PP2A in cerebral ischemia. The target organ of this study is brain tissue. PP2A subunit B is specifically expressed in brain tissues [19]. Thus, we focused on the expression of PP2A subunit B because it is more specific to brain tissue and also plays an important role in the development of neurons. We proposed that the reason for the significant change in subunit B during brain damage was its abundant expression in brain tissue that contributed to the performance of neuronal function. Western blot analysis was used to confirm the decrease of PP2A subunit B protein in MCAO animals. EGCG mitigates this reduction due to MCAO damage. The results of Western blot are similar to those of reverse transcription PCR. In addition, immunohistochemistry staining represents similar patterns in previous results, including reverse transcription PCR and Western blot analysis.

PP2A regulates the cell cycle and acts as a regulator of cell growth and development, signaling pathways, and cell mobility [14–16]. It can protect neurons against advances in neurodegenerative diseases because it can dephosphorylate hyperphosphorylated tau proteins [27, 28]. Downregulation of PP2A is associated with abnormal tau phosphorylation aggregation in Alzheimer’s disease [29, 30]. PP2A regulates Bcl-2 and Bad levels and prevents cell death from harmful stimuli [31, 32]. PP2A is also related with neurofibrillary tangle formation that causes neurodegenerative disorders [33]. We identified a decrease in PP2A subunit B through a proteomic approach [25]. We have previously reported significant declines in PP2A subunit B in focal cerebral ischemia and glutamate-exposed cell death [34, 35]. These findings reveal the importance of PP2A and its role in preventing cell death and neurodegenerative diseases. In addition, flavonoid chemicals regulate PP2A expression and regulate the apoptotic signaling pathway [36]. EGCG regulates adipogenesis through activation of the PP2A enzyme [37]. EGCG has neuroprotective effects and prevents cell death and neurodegeneration in Parkinson’s disease and Alzheimer’s disease [6–9]. EGCG prevents tumor progression due to activation of the PP2A signaling pathway [38, 39]. In addition, activation of PP2A by EGCG can improve the production of endothelial nitrogen oxides, which act as mediators of various cardiovascular functions [40]. These studies suggest that EGCG exhibits various biological functions through activation of PP2A. However, regulation of PP2A by EGCG in ischemia has not been reported. Our results clearly demonstrate that EGCG improves neurological deficits caused by MCAO damage and restores behavioral disorders. EGCG also alleviates the reduction of PP2A subunit B by MCAO damage. PP2A subunit B serves as a modulator of neuroprotection in ischemic brain damage.

## Conclusion

This study shows that EGCG alleviates neurological deficits and neuropathological lesions caused by focal cerebral ischemia. EGCG treatment also attenuates the reduction of PP2A subunit B due to ischemia. These results suggest that EGCG is involved in neuroprotective mechanism through regulation of PP2A subunit B in a stroke animal model. Therefore, this study provides evidence that EGCG can be used clinically as a neuroprotective agent for stroke.

## Methods

### Experimental animals and drug treatment

For this study, we purchased male adult Sprague Dawley rats (*n* = 48, weight 220–230 g) from Samtako Co. (Animal Breeding Centre, Osan, Korea). Rats were kept in an animal room where temperature and light (25 ^o^C, 12 h light/12 h dark cycle) were controlled for a week to adapt to the new environment and reduce stress before starting experiment. All experiments were completed with the provided guidelines from the Institutional Animal Care and Use Committee of Gyeongsang National University. Feed and water were freely given to all the animals and then randomly divided into four groups: vehicle + sham, EGCG + sham, vehicle + MCAO, and EGCG + MCAO group. EGCG (50 mg/kg, Sigma Aldrich, St. Louis, MO, USA) was dissolved in phosphate buffer saline (PBS) and injected into the abdominal cavity just before MCAO surgery. Animals in the vehicle group were injected with only PBS except EGCG.

### Middle cerebral artery occlusion

Animals were injected with 50 mg of Zoletil (Virbac, Carros, France) to induce anesthesia before MCAO surgery. They were carefully removed from the animal cage and placed on a heating pad to maintain normal body temperature during MCAO procedures. MCAO surgery was performed in accordance with the procedures previously described [41]. The midline of neck was incised, and the surrounding skin and muscles were opened to expose the right common carotid artery (CCA). CCA was carefully separated from surrounding tissues and nerves, and the right external carotid artery (ECA) and right internal carotid artery (ICA) were continuously exposed. CCA was fixed with a microvascular clamp to temporarily block blood supply through the CCA. The proximal end of the ECA was tied with a suture and cut. A nylon suture (4/0, nylon filament) with rounded tip by heating was inserted into the ECA and extended to the ICA until resistance was felt. The inserted nylon was tied with ECA using black silk to fix the nylon in place. The neck was closed with black silk and animals were carefully kept back in their cages. After 24 h MCAO surgery, animals were sacrificed and brain tissues were collected for further experiments.

### Neurological deficit scoring test

The neurological deficit score test was performed in accordance with the guidelines previously described [42]. Five-point scale was used for the test: 0, animals with normal posture; 1, animals with contralateral forelimbs flexion; 2, animals turning to one side; 3, animals leaning toward the affected side and seziures; 4, animals with no locomotor activity.

### Corner test

Corner test was used to evaluate the sensorimotor and postural asymmetries according to a previously described report [43]. Two whiteboards (30 × 20 × 1 cm^3^) were set with an angle of 30º including small space which induces animal to turn either right or left to exit the corner. Animals were allowed to enter the corner. When the vibrissae of animals were touched by the whiteboard, animal turn to right or left side. Test was repeated ten times on each animal and the number of right and left turns were recorded. Animals were trained for the corner test before MCAO surgery.

### Grip strength test

Forelimb strength was measured by performing grip strength test using a strength meter (Jeung Do Bio & Plant Co., Ltd., Seoul, Korea) as modification of the previously described method [44]. In each test, only one forepaw was tested, and the untested forepaw was temporarily wrapped with adhesive tape. Animals were allowed to grasp the bar and the gauge was reset to 0 g. They were slowly pulled back and the maximum force was recorded. The test was performed five trials and grip strength of each forelimb was recorded.

### Hematoxylin and eosin staining

Animals were sacrificed and brains were carefully removed from their skulls. The brains were cut into 50 mm thick sections and the cross sections were fixed in a 4% neutral buffered paraformaldehyde solution. The brain sections were washed with tap water overnight, dehydrated with graded ethyl alcohol series (70–100%) for 1 h, and then cleaned for 1 h with xylene. They were kept in a paraffin tank of the paraffin embedding center (Leica, Wetzlar, Germany). Brain tissue was embedded with paraffin and placed on a cooling plate to cement. The paraffin block was cut into a 4 μm thick section using a rotary microtome (Leica) and the paraffin ribbons were floated in tissue bath. The tissue ribbons were kept on slide glass side and dried on a slide warmer (Thermo Fisher Scientific, Waltham, MA, USA). The tissue slide was deparaffinized with xylene for 3 min and then rehydrated with graded ethyl alcohol series (100–70%). They were stained for 10 min in a Harris’ hematoxylin solution (Sigma-Aldrich) followed by washing with running tap water for 10 min. Tissue slides were differentiated in a 1% hydrochloric acid solution, dipped in water, neutralized in a 1% ammonia solution, and washed with water. They were stained with eosin Y solution (Sigma-Aldrich) for 1 min, dipped in the graded ethyl alcohol series (70 ~ 100%) to dehydrate, and then cleaned with xylene. A drop of permount mounting medium (Thermo Fischer Scientific) was dropped on the stained tissue and coverslipped. We observed the stained tissues using an Olympus microscope (Olympus, Tokyo, Japan). The image of the right cerebral cortex was captured and displayed in the results.

### Immunofluorescence staining

Brain tissue slides for immunofluorescence staining were prepared and rehydrated in the same manner as mentioned in hematoxylin and eosin staining. The deparaffinized slides were washed twice with PBS for 10 min, treated with proteinase K at room temperature for 5 min, and then washed twice again with PBS for 10 min. To prevent non-specific reaction, tissue slices were incubated with 5% normal goat serum for 1 h at room temperature. They were continuously incubated with anti-NeuN and anti-GFAP (diluted 1:100, Santa Cruz Biotechnology) overnight at 4 °C. The slides were washed twice with PBS for 10 min and treated with fluorescein isothiocyanate (FITC)-conjugated secondary antibody (dilution 1:100, Santa Cruz Biotechnology) for 90 min at room temperature. A drop of fluorescent mounting medium (Dako North America, Inc., Carpinteria, CA, USA) was dropped on a stained tissue and covered with cover glass. A fluorescence microscope (AXIO, Carl Zeiss Corporation, Thornwood, NY, USA) was used to observe NeuN and GFAP positive reactions. Images were captured from the cerebral cortex and selected in four random square areas (1 × 1 mm). The integrated density of NeuN and GFAP immunoreactivity was measured using Image J program (National Institute of Health, Bethesda, MD, USA).

### Reverse transcription-polymerase chain reaction (PCR)

The right cerebral cortex tissue was homogenized with Trizol Reagent (Life Technologies, Rockville, MD, U.S.A.) and total RNA was extracted according to the manufacturer’s manuals. Superscript III first-strand system (Invitogen, Carlsbad, CA, USA) was used to synthesize single stranded complementary DNA from 1 µg of total RNA samples. The target genes were amplified by polymerase chain reaction (PCR) using specific primers for each gene. Table 1 shows the primer used. The PCR process was conducted as following steps: denaturation for 5 min at 94 °C; 30 cycles of denaturation step at 94 °C for 30 s, annealing step at 54 °C for 30 s, and elongation step at 72 °C for 1 min; and a final extension for 10 min at 72 °C. PCR products were mixed with a Loading STAR dye (Dyne Bio, Seongnam, Korea), loaded on 1% agarose gel, and electrophoresed for 15 min using Mupid-2plus (Takara bio, Shiga, Japan). PCR products were visualized under an ultraviolet light, the images were captured and the intensity of the PCR bands was calculated using Image J program (National Institute of Health, Bethesda, MD, USA). Intensities levels were expressed as a ratio of PCR product density to β-actin density. The expression levels of gene were expressed as a ratio of PCR product intensity to β-actin intensity.

**Table 1:** Sequence of the primers used for PCR amplification

Gene	Primer sequences (F, Forward; R, Reverse)	Product (bp)
Protein phosphatase subunit A	F: 5’-AGTATGTGCACTGTCTGCTG-3’ R: 5’-GCAAACTGAGAAGAGACCAC-3’	212
Protein phosphatase subunit B	F: 5’-CCTGGTATGCCAAACTCGAT-3’ R: 5’-ACAATAGCCACCTGGTCGTC-3’	223
Protein phosphatase subunit C	F: 5’- GAGGCGAGCCACATGTCACT-3’ R: 5’- CCATTAGGTCAACAGACGGTGTT-3’	101
β-actin	F: 5’-TACAACCTTCTTGCAGCTCCTC-3’ R: 5’-CCTTCTGACCCATACCCACC-3’	205

### Western blot analysis

The right cerebral cortex tissue was isolated from the entire brain and homogenized in a lysis buffer [1% Triton X-100, 1 mM EDTA in 1 × PBS (pH 7.4)] containing 200 µM phenylmethylsulfonyl fluoride. The homogenate was sonicated for 3 min and centrifuged for 20 min at 15,000 × *g*. After centrifugation, the supernatant was transferred into a clean tube and the rest was discarded. The protein concentration of each sample was measured according to the manufacturer’s instructions using bicinchoninic acid (BCA) protein assay kit (Pierce, Rockford, IL, USA). Protein samples (30 µg) were loaded into 10% sodium dodecyl sulfate polyacrylamide (SDS-PAGE) gels already prepared for electrophoresis. Electrophoresis was carried out continuously until the blue dye reached the bottom of the gel. Gel was carefully removed from the glass and the protein of the gel was transferred to the polyvinylidene difluoride (PVDF) membrane using a semi-dry blotting system (ATTO Blotting System Corporation, Japan). PVDF membrane was reacted with 100% methanol for 3 min before transfer procedure. After transfer, the membranes were reacted with a 5% skim milk solution in tris-buffered saline solution with 0.1% Tween 20 (TBST) for 1 h at room temperature to prevent non-specific antibody bindings. PVDF membranes were washed with PBS three times for 10 min and treated with primary antibodies, anti-PP2A subunit B antibody (1:100, Cell Signaling Technology) and anti-β-actin antibody, at 4 °C overnight (diluted 1:1000, Cell Signaling Technology, Beverly, MA, USA, Santa Cruz Biotechnology, Santa Cruz, CA, USA). The next morning, PVDF membranes were washed with PBS three times for 10 min and incubated with a secondary antibody (anti-mouse IgG or anti-rabbit IgG, diluted 1:5000, Cell Signaling Technology) at room temperature for 2 h. Membranes were washed with TBST three times for 10 min and reacted with chemiluminescence detection reagents (GE Healthcare, Little Chalfont, BuckingChamshire, UK) for 1 min. They were exposed on X-ray film (Fuji film, Tokyo, Japan) for 1 min, X-ray film was developed in the developer and fixed in the fixed solution. X-ray films were dried and scanned, the intensity for detected band were analyzed by Image J program (Media Cybernetics, Rockville, MD, USA). The results were expressed as a relative intensity of PP2A subunit B band to the intensity of β-actin band.

### Immunohistochemical staining

After euthanasia, the whole brain was carefully removed from the skull, the middle region of the brain was cut to 50 mm thick and fixed in a 4% neutral buffered paraformaldehyde solution (pH 7.4) for 24 h. The brain tissues were removed from paraformaldehyde solution and washed with running tap water overnight. They were dehydrated with graded ethyl alcohol series (70–100%) for 1 h each step, and then cleaned with xylene. The brain tissue was kept for 1 h in the paraffin tank of the paraffin embedding center (Leica, Wetzlar, Germany) and embedded with paraffin. The paraffin blocks were cut into 4 μm thickness using a rotary microtome (Leica, Wetzlar, Germany). The sections were mounted on glass slides and dried on the slide warmer (Thermo Fisher Scientific). They were deparaffinized in xylene for 3 min, rehydrated with graded ethyl alcohol (100–70%), and washed with tap water. The sections were dipped in a 10 mM sodium citrate buffer (pH 6.0), microwaved for antigen retrieval, and then incubated with a 1% hydrogen peroxide solution in methanol for 10 min to inhibit the endogenous peroxidase activity. They were incubated with normal goat serum for 1 h to block non-specific binding and normal goat serum were removed. The sections were incubated with anti-PP2A subunit B antibody (1:100, Cell Signaling Technology) overnight at 4 °C. They were washed with PBS, incubated with biotinylated goat anti-rabbit IgG (1:200 in PBS) for 2 h, washed again with PBS, and then reacted with avidin-biotin-peroxidase complex for 1 h using a Vector ABC Elite kit (Vector Laboratories Inc., Burlingame, CA, USA). They were washed with PBS and stained with 3, 3′-diaminobenzidine tetrahydrochloride (DAB, Sigma-Aldrich) for 10 min. DAB reaction was performed until the color of the tissue was brown after 0.03% hydrogen peroxidase treatment. The sections were counterstained with hematoxylin solution (Sigma, Aldrich) for 3 min, rinsed with water, dehydrated with graded ethyl alcohol (70–100%), and cleaned with xylene. They were mounted with a drop of the permount mounting medium (Thermo Fisher Scientific, Walthem, MA, U.S.A) and covered with coverglass. The stained tissues were observed using an Olympus microscope (Olympus, Tokyo, Japan) and the images were taken in the right cerebral cortex. The number of PP2A subunit B positive cells was calculated and the level of PP2A subunit B expression was presented as a percentage of the number of PP2A subunit B positive cells to the number of total cells.

### Statistical analysis

All experimental data were represented as the mean ± standard error of means (S.E.M.) and the differences between groups were analyzed by two-way analysis of variance (ANOVA) followed by post-hoc Scheffe’s test. A P value of less than 0.05 (P < 0.05) was considered to be statistically significant. *P < 0.05.

## Data Availability

The data that support the findings of this study are available on request from the corresponding author on reasonable request.

## Abbreviations

BCA: Bicinchroninic acid
CCA: Common carotid artery
DAB: 3,3′-Diaminobenzidine
ECA: External carotid artery
EGCG: Epigallocatechin gallate
GFAP: Glial fibrillary acidic protein
ICA: Internal carotid artery
MCAO: Middle cerebral artery occlusion
NeuN: Neuronal nuclear protein
PBS: Phosphate-buffered saline
PCR: Polymerase chain reaction
PP2A: Protein phosphatase 2 A
PVDF: Polyvinylidene difluoride
ROS: Reactive oxygen species
RT-PCR: Reverse-transcript polymerase chain reaction
SDS-PAGE: Sodium dodecyl sulfate polyacrylamide
TBST: Tris-buffered saline containing 0.1% Tween-20
